# Bringing systems biology to cancer, immunology and infectious disease

**DOI:** 10.1186/s13059-014-0407-1

**Published:** 2014-07-31

**Authors:** Kevin A Janes, Chun-Chao Wang

**Affiliations:** Department of Biomedical Engineering, University of Virginia, Charlottesville, VA 22908 USA

**Keywords:** Single cell, Heterogeneity, Model, Microscopy, Mass cytometry, Cytokine

## Abstract

A report on the seventh annual ‘International Conference on Systems Biology of Human Disease’ held in Boston, Massachusetts, USA, 17–19 June, 2014.

Over 250 scientists converged upon Harvard Medical School for the seventh annual International Conference on Systems Biology of Human Disease (SBHD). Originally conceived by the systems-biology working groups of Boston and Heidelberg, the SBHD has grown to become an important venue for disease-relevant research at the systems level. The modest size of SBHD hits that conference sweet spot, where you can still make five or six new friends while touching base with 15 or so old ones.

SBHD dedicates itself to systems biology, a field that is in its teenage years as a discipline. Like most teenagers, systems biology no longer yearns for the approval of its parents (molecular biology and mathematics), even though it cannot possibly succeed on its own without them. We get caught up as a group in the latest fashions (cellular heterogeneity), whereas others fall out of style (interaction hairballs). Fortunately, the upbringing of the systems-biology community has been healthy so far - cliquishness is at a minimum, and good science is at the forefront. During the meeting, we heard from cell biologists, engineers, geneticists, theoreticians, technologists and bioinformaticians, who all connected with systems biology in various ways. There were also multiple invited talks on microbiology, an area that has been somewhat slower to adopt systems approaches outside of model organisms.

## Mining cell-to-cell heterogeneity

Heterogeneity pervaded the meeting so much that SBHD could have stood for ‘single-cell biology and human disease’. Appreciating cell-to-cell heterogeneity is pretty straightforward - just look at cells with a microscope and you will see that no two are identical. But, do we know how different they truly are? Chris Bakal (Institute of Cancer Research, London, UK) asked this question with respect to cell shape and its impact on cell signaling. Focusing on signaling through nuclear factor-κB (NF-κB), Bakal found that the strongest responders in a population exhibited unique nuclear-shape characteristics compared with those of the average. Cell density played an important role, as cells at the leading front of a collectively migrating sheet showed preferential NF-κB activation in response to tumor necrosis factor. A more complete inventory of shape-sensitive pathways would be valuable for interpreting high-content imaging screens with such information already embedded.

Equally striking were the live-cell examples of cell-to-cell heterogeneity. Using modern Förster resonance energy transfer (FRET) reporters of extracellular signal-regulated kinase (ERK) and 5'-AMP-activated protein kinase (AMPK) activity, John Albeck (University of California Davis, USA) showed time-lapse videos of how the single-cell response to environmental stimuli can differ qualitatively from the population average. Pulses of activity were observed over many hours, with time-dependent characteristics that depended on the sensor and the perturbation. Understanding how cells interpret these pulses will require computational models that combine data on signaling, gene expression and post-translational modifications of immediate-early gene products.

FRET-based indicators of kinase activity can be problematic for multi-color applications and for kinases with rapid deactivation kinetics. To address these limitations, Sabrina Spencer (University of Colorado Boulder, USA) and Markus Covert (Stanford University, USA) presented independent designs of one-color sensors that report kinase activity by localization. The trick is to engineer substrates within a tandem nuclear localization and export sequence, such that phosphorylation disrupts the more-potent sequence and causes relocalization of the reporter. Spencer designed a reporter for cyclin-dependent kinase 2 in order to investigate the cellular decision to proliferate or quiesce, revealing a new restriction point in G_2_ phase that had eluded the ‘starve-and-refeed’ experiments of 40 years ago. Covert expanded the premise of ‘kinase translocation reporters’ (KTRs) more broadly to the mitogen-activated protein kinases (MAPKs). Covert showed proof-of-concept multiplexing of one-color KTRs by tracking ERK, c-jun N-terminal kinase (JNK) and p38 activities concurrently in single cells. KTRs showed better reversibility than standard FRET reporters, suggesting that they could become the sensor of choice for pathways that are rapidly deactivated.

Bernd Bodenmiller (University of Zürich, Switzerland) took deep analysis of static images to the next level with imaging mass cytometry. In Bodenmiller’s setup, tissue sections are immunostained with heavy metals and then raster ablated with a UV laser before detection of the released metals by mass spectrometry. Work in progress seeks to identify intermediate states during the epithelial-to-mesenchymal transition of mammary cancer cells. The sensitivity of imaging mass cytometry should improve substantially as the technology matures.

## Immune cells and cytokine signaling

Another recurring theme at SBHD 2014 was systems analysis of the immune system and circulating cytokines. We crossed over from epithelial to immunological heterogeneity with talks from Kathryn Miller-Jensen (Yale University, USA) and Grégoire Altan-Bonnet (Memorial Sloan-Kettering Cancer Center, USA) about single-cell responses of myeloid and lymphoid effectors. Miller-Jensen reported on the cascade of paracrine factors triggered by lipopolysaccharides in monocytes and macrophages. Using nanowell chips to capture and profile cytokines released from single cells, Miller-Jensen compared the population-level secretion patterns with those obtained from individual cells isolated from the population. Several late-phase cytokines - including interleukin-6, interleukin-10 and granulocyte-macrophage colony-stimulating factor - appeared to be emergent properties of the population that could not be recapitulated by the aggregate response of individual cells. Although Miller-Jensen focused on a bacterial stimulus, these results could be especially relevant to solid tumors and atherosclerosis, where macrophage accumulation is recognized.

Altan-Bonnet embraced the intersection of cancer biology and immunology by investigating the receptor-proximal behavior of transformed B cells in chronic lymphocytic leukemia (B-CLL). Comparing B-CLL tyrosine kinase signaling with that of B cells from healthy donors, he reported bimodal and hysteretic responses of B-CLL cells to inhibition of tyrosine phosphatases. Altan-Bonnet tied these dynamical-systems properties of B-CLL cells to aberrant B-cell receptor clustering, which gives rise to cooperativity and a saddle-node bifurcation of the network. This mechanism provides a potential explanation for why B-CLL cells escape negative selection, and the bimodality itself could be exploited as a sensitive diagnostic for staging B-CLL patients.

Of course, good systems biology is still taking place at the population level for blood cells and their signaling pathways. Ursula Klingmüller (German Cancer Research Center, Heidelberg, Germany) examined the potential dangers of erythropoietin (Epo) therapy for treating anemia in lung cancer patients undergoing chemotherapy. Certain non-small cell lung cancers (NSCLCs) can express Epo receptors at very low levels, causing chemoresistance. By combining modeling with assay development, Klingmüller showed that NSCLCs consume Epo even though they may possess only approximately 50 receptors on their cell surface. Moreover, she showed that not all Epo variants were equivalently bioactive towards NSCLCs and erythroid progenitors, suggesting that some variants might be better suited for chemotherapy-induced anemia than others.

Cytokine crosstalk was an important motivation for the systems work on endometriosis presented by Douglas Lauffenburger (Massachusetts Institute of Technology, USA). By monitoring the cytokine profiles of aspirates of peritoneal fluid from women stricken with the disease, Lauffenburger showed how one could infer the secreting and receiving cell types that were most consistent with the observed profiles. This analysis suggested the existence of macrophage hyperactivity and JNK signaling in endometriosis patients with cytokine signatures that correlated to pain and emphasized the practical constraints that must be considered when combining systems biology with clinical material.

## Infectious disease

The latest entries into the systems-biology arena at SBHD 2014 involved the bacteriology of infectious disease. Pathway and network models are a long way off because most genes lack detailed functional characterization, and it is not even clear which ones are essential under different conditions. Christopher Sassetti (University of Massachusetts Medical School, USA) and Tim van Opijnen (Boston College, USA) tackled this problem for *Mycobacterium tuberculosis* and *Streptococcus pneumoniae* at the genomic level. Using different insertional-mutagenesis and sequencing-based approaches, Sassetti and van Opijnen showed how ‘conditional essentiality’, dictated by nutrient availability and other stresses in the host microenvironment, could be the norm for these infectious agents. Versatile pathogens have not one network but many that reconfigure according to the growth conditions. A systems-level dissection of the constraints on these networks might investigate whether bacteria could be ‘trapped’ in certain configurations that eradicate infection.

## Concluding remarks

Amidst all the great things brought to SBHD 2014, it was striking to note what was missing. For example, aside from our talks and that of Luis Serrano (Center for Genomic Regulation, Barcelona, Spain), no presentation showed results with quantitative immunoblots, as if systems biology had disowned one of its parents. Indeed, Serrano showed that immunoblots were more robust for absolute protein quantification than multiple reaction monitoring, a mass-spectrometry-based approach that is currently in vogue. Diverse perspectives imply a diversity of methods that intermingle experimental and computational techniques, both old and new. Like teenagers, we want nothing but to race around in a fast sports car, forgetting that we must still use two legs to walk to the driver-side door.

## Abbreviations

AMPK: 5'-AMP-activated protein kinase
B-CLL: B-cell chronic lymphocytic leukemia
Epo: Erythropoietin
ERK: Extracellular signal-regulated kinase
FRET: Förster resonance energy transfer
JNK: c-jun N-terminal kinase
KTR: Kinase translocation reporter
NF-κB: Nuclear factor-κB
NSCLC: Non-small cell lung cancer
SBHD: Systems Biology of Human Disease

